# Realist synthesis of intentional rounding in hospital wards: exploring the evidence of what works, for whom, in what circumstances and why

**DOI:** 10.1136/bmjqs-2017-006757

**Published:** 2018-03-14

**Authors:** Sarah Sims, Mary Leamy, Nigel Davies, Katy Schnitzler, Ros Levenson, Felicity Mayer, Robert Grant, Sally Brearley, Stephen Gourlay, Fiona Ross, Ruth Harris

**Affiliations:** 1 Florence Nightingale Faculty of Nursing, Midwifery and Palliative Care, King’s College London, London, UK; 2 School of Health, Sport and Bioscience, University of East London, London, UK; 3 Kingston Business School, Kingston University, London, UK; 4 Independent Researcher, London, UK; 5 Nurse Development Team, South West London and St George’s Mental Health NHS Trust, London, UK; 6 Centre for Health and Social Care Research, Kingston University and St George’s University of London, London, UK

**Keywords:** healthcare quality improvement, health services research, nurses, patient safety

## Abstract

**Background:**

Intentional rounding (IR) is a structured process whereby nurses conduct one to two hourly checks with every patient using a standardised protocol.

**Objective:**

A realist synthesis of the evidence on IR was undertaken to develop IR programme theories of what works, for whom, in what circumstances and why.

**Methods:**

A three-stage literature search and a stakeholder consultation event was completed. A variety of sources were searched, including AMED, CINAHL, MEDLINE, PsycINFO, HMIC, Google and Google Scholar, for published and unpublished literature. In line with realist synthesis methodology, each study’s ‘fitness for purpose’ was assessed by considering its relevance and rigour.

**Results:**

A total of 44 papers met the inclusion criteria. To make the programme theories underpinning IR explicit, we identified eight a priori propositions: (1) when implemented in a comprehensive and consistent way, IR improves healthcare quality and satisfaction, and reduces potential harms; (2) embedding IR into daily routine practice gives nurses ‘allocated time to care’; (3) documenting IR checks increases accountability and raises fundamental standards of care; (4) when workload and staffing levels permit, more frequent nurse–patient contact improves relationships and increases awareness of patient comfort and safety needs; (5) increasing time when nurses are in the direct vicinity of patients promotes vigilance, provides reassurance and reduces potential harms; (6) more frequent nurse–patient contact enables nurses to anticipate patient needs and take pre-emptive action; (7) IR documentation facilitates teamwork and communication; and (8) IR empowers patients to ask for what they need to maintain their comfort and well-being. Given the limited evidence base, further research is needed to test and further refine these propositions.

**Conclusions:**

Despite widespread use of IR, this paper highlights the paradox that there is ambiguity surrounding its purpose and limited evidence of how it works in practice.

## Introduction

In 2013 the Francis Inquiry^1^ examined evidence about the reasons for failures in patient care at Stafford Hospital, UK, and made recommendations for improvement. One of these recommendations was that ‘regular interaction and engagement between nurses and patients and those close to them should be systematised through regular ward rounds’ (p1510).^1^ This recommendation has become synonymous with ‘intentional rounding’ (IR), a structured process developed in the USA and marketed by the Studer Group,^2^ whereby nurses carry out one to two hourly checks with every patient using a standardised protocol and documentation (box 1).

Box 1 **Typical intentional rounding schedule in acute ward settings^2 5^**

During each round, the following standardised protocol is used by a nurse for each patient:

- An opening phrase is used by the nurse to introduce his or herself and to put the patient at ease.
- Scheduled tasks are then performed.
- A discussion of the four key elements of the round, often called the ‘4 P’s’:
  - Positioning—making sure the patient is comfortable and assessing the risk of pressure sores.
  - Personal needs—assessing patients’ personal needs, including whether they need assistance with getting to the toilet.
  - Pain—asking patients to rate their level of pain on a scale of 0–10.
  - Placement—ensuring any items a patient needs are within easy reach.
- An assessment of the care environment, such as checking the temperature of the room or any fall hazards.
- Ending the interaction with a closing phrase such as “Is there anything else I can do for you before I go?”.
- The patient is informed of when the nurse will return.
- The nurse documents the round.

If patients are unable to respond during the round, the nurse may follow this process with family members.^61^

Box as cited in ref 15.

The UK government’s response to the Francis report expected all National Health Service (NHS) hospitals to implement IR within 1 year,^3 4^ reinforced by a ‘comply or explain’ approach used in Care Quality Commission hospital inspection,^4^ and as a result IR has been introduced in the majority of hospitals. A number of benefits have been claimed, including a reduction in call bell use, falls and pressure sores, and increased patient satisfaction.^2 5–8^ However, substantial limitations to this evidence base have been highlighted, including concerns around selection bias, potential conflict of interest, and weaknesses in study design and analysis,^3 9 10^ which may, in part, be due to what Marshall *et al* 11 would say is the urge to act combined with the absence of a scientific approach to improvement science.^12^ In line with recommendations for evaluative approaches in improvement science advocated by Don Berwick,^13^ for this study we adopt a realist evaluation^14^ approach to investigate the impact of IR in hospital wards on organisation, delivery and experience of care.^15^ For the first phase of this evaluation, a realist synthesis of IR was undertaken with the view to develop programme theories to provide a framework for the analysis.

Box 2 **Definitions of realist terms and how they have been used in this review**

Context (C): This refers to ‘the ‘backdrop’ of programmes and research…broadly understood as any condition that triggers and/or modifies the behaviour of a mechanism’ (p317).^62^ Mechanism (M): ‘…mechanisms are underlying entities, processes or structures which operate in particular contexts to generate outcomes of interest’.^63^ More specifically, ‘…mechanisms are a combination of resources offered by the social programme under study and stakeholders’ reasoning in response. 14

Outcome (O): The outcome is a result of the interaction between a mechanism and its triggering context.

Programme theory: The programme theory specifies what is supposed to be done in a policy or programme (theory of action) and how and why that is expected to work (theory of change).^18^

Realist synthesis is a theory-driven approach to evaluating complex social interventions such as IR, using empirical evidence from the literature.^16 17^ Realist synthesis seeks to understand for whom the intervention works and does not work, how, why and in what circumstances.^17–19^ It does this by identifying the relationships between particular contexts, mechanisms and outcomes (known as ‘context-mechanism-outcome configurations’ or ‘CMOs’) at a granular level, which explain an intervention’s successes and failures.^16 18 20^

As available theory on the potential of IR was limited, the aim of this realist synthesis was to help develop programme theories of IR to generate hypotheses around for whom IR may or may not work, in what circumstances and why.

## Methods

Given that this was the first phase of a larger realist evaluation study, realist synthesis was deemed to be the most appropriate method to use for the initial period of theory development, as it applied a realist approach to retrospective literature reviewing. The realist synthesis was undertaken following practical ‘how-to’ guidance in the field,^17 18^ and the authors ensured that quality and publication standards in realist synthesis were upheld.^21 22^ It is acknowledged within the field of realism that a number of different conceptualisations or definitions of ‘mechanisms’ have been identified.^18^ Box 2 provides definitions of how we have used the Context, Mechanism, Outcome and Programme theory terminology in this review.

The realist synthesis was undertaken using a three-stage literature search and a stakeholder consultation event. In stage 1, a search of academic, policy and grey literature was undertaken to develop initial programme theories of IR (ie, purported ideas of ‘what is supposed to happen?’ or ‘how is it supposed to work?’).^18^ Expert advice was sought from library and information sciences specialists around generating relevant search terms. Between June and July 2014, four electronic databases were searched (Allied and Complementary Medicine Database (AMED), Cumulative Index of Nursing and Allied Health Literature (CINAHL), Medical Literature Analysis and Retrieval System Online (MEDLINE), Royal College of Nursing Archive (RCN Archive), alongside searches of Google, Google Scholar, InterNurse, Science Citation Index Expanded (SCIE) and NHS Evidence, using the strategies highlighted in box 3.

Box 3 **Search strategy**

**Strategy for searches in Allied and Complementary Medicine Database (AMED), Cumulative Index of Nursing and Allied Health Literature (CINAHL), Medical Literature Analysis and Retrieval System Online (MEDLINE), Royal College of Nursing Archive (RCN Archive), Psychological Information Database (PsycINFO), Health Management Information Consortium (HMIC), Excerpta Medica Database (Embase), Scopus, British Medical Journal Journals (BMJ Journals) and the Cochrane Library**

**Free text terms and operators**

“Intentional round*” OR “hourly round*” OR “patient round*” OR “purposeful round*” OR “nursing round*” OR “comfort round*”

AND

“nurs*”

(**Limiters**: abstract only (where applicable), English-language only)

**Strategy for searches in Google, Google Scholar, InterNurse, SCIE and NHS Evidence (each search undertaken separately)**

“Intentional round”

“Hourly round”

“Patient round”

“Purposeful round”

“Nursing round”

“Comfort round”

Eighty-nine relevant documents were identified and divided between two researchers. One researcher read all the empirical research papers (n=42) and the other read all the grey literature and policy documents (n=47). Both independently examined their documents to identify any purported mechanisms of IR (ie, theories or assumptions about why/how IR worked/was expected to work). Each wrote a brief description of the mechanism based on what they had found and recorded the names of the papers in which they were identified. The researchers then met to discuss their findings. Eight potential mechanisms were identified in the empirical research papers, and six of these mechanisms were also identified in the policy documents (see table 1). Where both researchers found a similar mechanism, they each briefly described what they had found and combined these descriptions together to produce the mechanism names and joint definitions. When mechanisms were only identified by one researcher, the other also read the paper(s) in which they had been identified and provided their input into the mechanism name and description. Through consensus, a combined list of eight mechanisms of IR was produced (table 1). A similar process was undertaken for context (table 2) and outcomes (table 3).

**Table 1:** Hypothesised mechanisms of intentional rounding (stage 1)

Mechanism title	Mechanism (resources)	Mechanism (reasoning/responses)
M1: Consistency and comprehensiveness*	Intentional rounding helps keep patient care consistent through the use of a structured, systematic approach, ensuring all patient needs are met and potentially less obvious aspects of care are considered and managed at every round. Intentional rounding also helps ensure that family members are provided with consistent care and information in line with their needs (eg, the need for information, to be respected and to be comforted). It can also prompt agency staff to deliver care to a required standard.	This provides reassurance and confidence in the quality of care to patients, their family members and staff.
M2: Allocated time*	Intentional rounding gives nurses allocated ‘time to care’ (ie, time to check that patients are comfortable and their needs are being met, thereby treating patients with dignity and replaces ‘presumed care’).	This helps nurses to organise their work and feel able to prioritise this aspect of nursing care.
M3: Accountability*	Staff are required to complete and sign the intentional rounding record to say they have carried out hourly checks.	This makes staff feel personally accountable for the standard of care. This enables ward managers to monitor and audit the standard of care provided by nursing staff.
M4: Nurse–patient relationships and communication*	Intentional rounding provides increased and improved communication between staff, patients and family members, and ensures that patients’ perceived basic fundamental needs are met. It also provides more opportunities for positive nurse–patient relationships to develop based on trust, respect and caring.	This enables staff to get to know patients better and become more aware of their needs, notice unusual behaviours/appearances and detect subtle/significant changes that can impact on comfort and safety.
M5: Visibility*	Intentional rounding increases the visibility/presence of nurses within a unit by increasing the time that nurses spend in the direct vicinity of their patients (ie, it gets nurses to the patient’s bedside).	This relieves the uncertainty and anxiety often associated with vulnerable patients’ hospital experience (ie, the inability to predict when care will be delivered and when someone will be available to assist them with care). This is comforting to family members because it denotes frequent and continuous assessment of the patient and their needs.
M6: Anticipation*	Intentional rounding enables nurses to anticipate/pre-empt and proactively address patient needs instead of being reactive and waiting for patient call bells and alarms.	This ensures that all patients receive regular care instead of unequally distributed care among patients, focused towards those who have frequent call bell use.
M7: Staff communication and/or teamworking	Intentional rounding provides healthcare professionals with documented evidence.	This is used to enhance staff communication and teamwork, and prioritise care in future rounds.
M8: Patient empowerment	Intentional rounding provides an opportunity for nursing staff, patients and family members to get to know each other better.	This empowers patients to ask for what they need in order to maintain their comfort and well-being.

NB: All of the mechanisms were identified in empirical research papers, and six (marked with *) were also identified in the grey and policy literature.

**Table 2:** Influencing contexts of intentional rounding (IR)

Context	Description
	**Individual capabilities and characteristics of key actors**
Staff education, training and understanding of IR (n=28)^6 8 25–27 31–33 36 39 41–45 47–52 54 64–69^	Staff education, understanding and training in IR were commonly viewed as an important factor in its success.^6 8 25 27 31 32 36 39 41 43–45 47 50 52 54 65^ Methods of educating staff about IR varied, including lectures/presentations, ward meetings, online learning modules, competency tests and feedback to staff on practice.^25 26 32 36 42 43 49 51 64–69^ Even where substantial education/training opportunities were available, not all managers participated or enabled their team to participate,^31^ and some staff remained unprepared for IR.^47^ Participating in IR educational opportunities was more difficult for nurses working night shifts^33^ and for agency/‘floating’ nurses.^54^
Staff engagement and motivation (n=23)^6 8 25 26 29 31–33 35–38 40–43 47 48 50–52 65 70^	IR was more successful when staff were engaged with, committed to and positive towards IR from the outset and throughout.^6 8 26 29 36–38 40 41 43 48 51 52 70^ Methods of fostering engagement varied, including involving staff in the design and implementation of IR initiatives, sharing best practice and developing a strong sense of team.^8 25 31 36 38 42 43 48 51^ IR was implemented less successfully when staff were resistant towards it, perceived it as a top-down process/paper exercise or did not believe it improved patient experience: in these situations IR was performed irregularly/with little intention by staff and poor activity not routinely challenged.^26 33 35 38 47 50^
Staff characteristics (n=2)^38 40^	Understanding of the principles and practices of IR varied according to individual staff characteristics, including age and level of training/experience.^38 40^
Leadership/management support for IR (n=18)^6 24 31 33 34 38 42 43 45 47 48 51 52 54 64 65 68 70^	Support from nursing leadership/management was key to successful IR, with leaders being required to provide a number of functions, including encouraging staff ‘buy in’, providing ongoing reminders and tips for success, and monitoring performance.^6 24 38 42 43 45 47 48 51 52 64 68 70^ Some papers highlighted the importance of unit champions/rounding experts/‘buddy support’,^6 38 47 54 65^ although others did not acknowledge their value.^33^ Senior ‘walkabouts’ were seen as useful,^38^ but some reported variable long-term commitment of leaders towards IR.^31^
Type of patients (n=15)^6 28 31 33 35 37 38 40–42 44–46 48 50^	Nurses did not think all patients required hourly rounding, and some patients did not want to be seen every hour.^6 31 33 35 37 38 41 42 45 46^ Complex and demanding patients took up more of nurses’ time during IR and were prioritised over those deemed to be ‘well’.^6 28 44 48 50^ IR was highlighted as most beneficial for older and vulnerable patients who required help with activities of daily living.^37^
Patient and carer education and understanding of IR (n=1)^67^	Variations in the amount of education and information around IR that patients and carers receive do not appear to have a significant impact on their perceptions of care.^67^
	**Ward characteristics**
Ward setting/layout (n=5)^25 34 37 38 42^	IR may be less suitable/more difficult to implement in some settings, including Accident and Emergency (A&E) and mental health.^25 37 38 42^ Open ward layouts facilitate IR, while closed or cluttered layouts inhibit it.^34^
Workload issues/lack of time (n=13)^6 28 30 31 34 35 37 40 44 47 50 53 69^	IR was inhibited when nurses faced competing tasks and priorities in their workload caused by busy wards, staff shortages, poor skill mix, high patient to nurse ratios, interruptions, emergencies or high numbers of complex patients.^6 28 30 31 34 35 37 40 44 47 50 53 69^
Who conducts the rounds? (n=6)^7 34 37 38 40 42^	There was variation across studies around who delivered IR (ie, staff of all levels vs senior staff only).^7 34 38 40 42^ Confusion around who should be delivering IR was a barrier to its implementation.^40^ IR worked best when all types of nursing, care and support staff were involved but where senior staff retained an active and visible daily role.^38^
	**Organisation and system characteristics**
Design and suitability of IR documentation (n=14)^28 29 32 35 37–39 41–43 45 50 51 53^	Staff who did not acknowledge the benefits of IR documentation or believed this to be a burdensome, ‘tick-box exercise’ perceived the IR process negatively.^28 35 38 45 50 53^ Frustration occurred when IR paperwork duplicated information recorded on other charts/logs.^28 39 42 53^ To document IR effectively, personalised tools were required to suit the setting and needs of the ward.^29 32 38 41–43 51^
Presence of other organisational changes/competing initiatives (n=6)^31 43 45 46 48 53^	Introducing multiple, simultaneous initiatives or changes alongside IR had a negative impact on its implementation.^31 43 45 46 48 53^
Embedding into existing daily routines (n=5)^6 8 31 38 47^	Successful implementation of IR required cultural change within organisations.^6 31 38 47^ Some highlighted the importance of embedding IR within existing daily routines,^31^ but others felt ward activity should be reorientated to fit around IR.^6^
Staged or simultaneous implementation approach (n=5)^6 26 34 38 52^	Variations in the implementation approach for IR were noted: some reported a staged introduction of IR,^6 26 38^ while others introduced it simultaneously across different wards and departments.^38^ The advantages of rolling out IR across the whole organisation outweighed the advantages of a more gradual approach.^38^
Reason for implementation (n=2)^31 53^	The reason behind the implementation of IR can influence staff perceptions of it. For example, the fact that IR was a government initiative helped some leaders to promote the concept in their clinical areas, but others reported resenting the intervention for the same reason.^31^

**Table 3:** General outcomes of intentional rounding (IR)

Outcome	Description	Expected change in outcome
	**Self-report patient and staff outcomes**	
Patient and carer satisfaction and perceptions of care (n=24)^6–8 24 25 27–29 36 40 42 46 48 49 54 64–69 71–73^	Nineteen studies reported IR increased patient and/or carer satisfaction or improved their perceptions of care,^6–8 24 25 27–29 36 40 42 48 49 64 65 68 69 71 73^ although some had small sample sizes, low response rates, methodological concerns or did not report findings to support their claims/state whether any differences were statistically significant.^28 36 65 71^ Four studies reported no statistically significant differences in patient and/or carer satisfaction or perceptions of care following IR implementation,^54 66 67 72^ and one found mixed results.^46^	Majority of studies reported IR *improved* patient and/or carer satisfaction.
Staff satisfaction and perceptions of care (n=9)^6 25 28 31 36 38 43 70 72^	Four studies reported IR improved staff perceptions of care provided and/or increased job satisfaction,^25 36 38 72^ and two associated IR with benefits to staff turnover.^36 43^ Two studies associated IR with more negative staff perceptions/experiences of care.^6 31^ One reported a statistically significant difference between nurses’ perceived benefits of IR for patients and for themselves, identifying IR as benefiting patients but not staff.^28^	Some evidence of *improved* job satisfaction and reductions in staff turnover
Patient uncertainty/anxiety (n=1)^69^	One study reported that IR reduced patient uncertainty around whether a caregiver would come to their assistance for immediate needs.^69^	One study reporting *reduction* in patient uncertainty
Patient awareness of IR (n=2)^35 38^	There was little evidence that patients were aware of the IR process, although most felt their needs were attended to on a regular basis or that they saw their nurse ‘enough’.^35 38^	Limited evidence
	**Clinical and management outcomes**	
Call bell use (n=18)^6 7 24–26 29 30 40 43 46 48 52 54 64–67 69^	Twelve studies reported a decrease in call bell frequency following the introduction of IR,^6 7 24 25 29 30 40 43 48 64 65 69^ although the same concerns around anecdotal reports and a lack of findings to support these claims were noted. Three studies reported an overall increase in call bell frequency following the introduction of IR.^54 66 67^ Others reported mixed results, such as variations in call bell usage across different wards within the same study.^26 52^ Two studies concluded IR did not reduce call bell frequency,^26 46^ but two noted a reduction in call bell duration.^6 26^	Majority of studies reported call bell use decreases with IR.
Pressure ulcers (n=8)^6 8 26 33 39 40 43 71^	Seven studies reported a decrease in the number of hospital-acquired pressure ulcers and/or improvements in the early detection of pressure ulcers following the implementation of IR (although again some of these studies had the methodological/reporting issues previously highlighted or were based on staff reports).^6 8 26 33 40 43 71^ One study reported mixed results around the impact of IR on pressure ulcers.^39^	Majority of studies reporting *decrease* in number of pressure ulcers
Pain management (n=3)^33 40 43^	Three studies reported improvements in patients’ pain management following the implementation of IR, although two were based on staff reports only^33 40^ and one did not provide findings to support these claims.^43^	Some evidence reporting *improvements* in pain management
Patient falls (n=22)^7 8 24–26 28 32–34 36 39 41 43 45 51 52 54 64 67 69–71^	Thirteen studies reported a decrease in the number of patient falls following the implementation of IR,^7 24–26 28 32–34 36 43 51 54 69^ although some were based on anecdotal staff reports/perceptions only^33 34^ or did not report findings to support their claims/state whether any changes were statistically significant.^26 28 32 36 43^ Five studies reported no statistically significant change in the overall falls rate^8 39 64 67 70^ and one reported an increase.^41^ One study found initial positive gains, although falls rates returned to baseline levels in a 1-year follow-up.^45^ Others said decreased falls rates could be attributed to other initiatives and not necessarily IR.^52 71^	Majority of studies reported *decrease* in patient falls.
Walking distances (n=2)^53 67^	One study reported increased walking distances for staff as a consequence of implementing IR,^53^ but another found more mixed results.^67^	*Mixed* findings
	**Health economic outcomes**	
Costs (n=1)^38^	No clear impact of IR on hospital costs has been highlighted.^38^	No impact
Patient readmission rates (n=1)^67^	No significant differences were found in 30-day patient readmission rates between the IR intervention and control groups in one study.^67^	No impact
	**Hospital-reported patient outcomes**	
Leaving without being seen/against medical advice (n=1)^25^	IR reduced the number of patients leaving A&E without being seen by 23.4% and leaving against medical advice by 22.6% in one study.^25^	One study found *reduction* in patients leaving against medical advice.
Patient complaints (n=5)^6 26 39 42 43^	Three studies reported patient complaints reduced after implementing IR, although this was either based on staff report only or findings were not reported to support this claim.^6 26 43^ The third study acknowledged the reduction in complaints could be attributed to other ongoing initiatives and not necessarily to IR.^26^ Two studies either reported no change in the overall number of patient complaints associated with IR or stated that complaints were too few in number to measure a notable difference.^39 42^	Majority of studies reported *reduction* in patient complaints.

Between October and November 2014, the stage 2 search was undertaken to refine programme theories by identifying empirical research that either supported or refuted them or identified any new ones. A comprehensive search for empirical research was undertaken in databases AMED, CINAHL, MEDLINE, PsycINFO, HMIC, Embase, Scopus, RCN Archive, InterNurse, BMJ Journals and the Cochrane Library using the search strategy highlighted in box 3. Snowball searches and citation searches in CINAHL and Scopus were also conducted. Nine new research papers were identified and combined with the 42 empirical research papers identified in stage 1. A bespoke structured data extraction form was completed for every paper, recording either salient details on the study design, objectives and participants, or the reason for its exclusion. Broad inclusion criteria were applied, meaning that if a paper described nursing rounds occurring every 2 hours or less and highlighted empirical evidence of any associated context, outcome or mechanism, it was included. Due to constraints in the study’s time and finances, it was necessary to focus the scope of the review on IR alone, rather than cross disciplinary boundaries or seek out data from alternative interventions, which might have the same mechanisms in operation.^17 22^ Conventional approaches to quality appraisal were not used and instead each study’s ‘fitness for purpose’ was assessed by considering its relevance and rigour.^16 23^ That is, in line with realist methodology, studies were not appraised by their study design but by whether or not they contributed to theory building and were credible and trustworthy.^17 18^

Thirty-five papers met the inclusion criteria and were included in the synthesis at this stage. The evidence collected from these papers was synthesised by drawing together all information on contexts, mechanisms and outcomes. Similarities and differences in findings were sought to build a comprehensive description of each mechanism and its role in IR. Due to the small number of papers identified for the ‘patient empowerment’ mechanism, focused literature searches specifically for this mechanism were also conducted, although no new papers were identified. A stakeholder consultation event was held in February 2015, involving service user representatives and NHS clinical staff (n=28), to discuss the mechanisms, contexts and outcomes arising from the synthesis, to ensure none had been missed, and to decide whether or not the researchers should focus only on particular mechanisms or to continue the synthesis with a broader focus.^22^ The stakeholders felt that the synthesis was appropriately focused to achieve optimal end-user relevance and that further refinement was not required at this stage. A third and final search of the literature was undertaken in February 2016 to ensure the synthesis was up-to-date and that no research published in the interim period had been missed. Stage 2 searches were repeated but focused only on research published between December 2014 and February 2016. Snowball searches and hand searches were undertaken and a further nine relevant papers were identified (44 papers in total, 1 early online). Data extraction forms were completed for each newly identified paper and any evidence added into the hypothesised CMOs.

## Results

**Figure 1:**
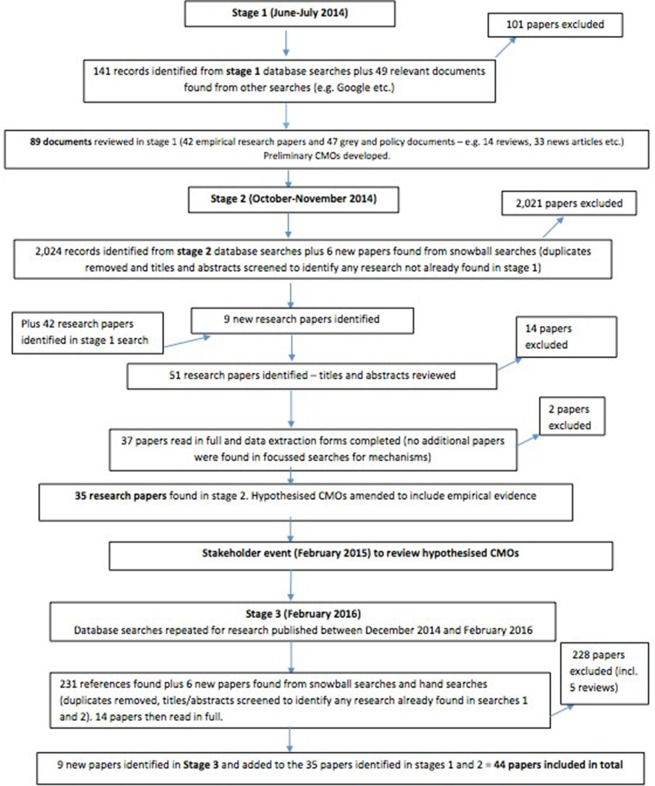
Document flow chart of the search process.

A document flow diagram for the synthesis process is provided in figure 1. The 44 papers were drawn from reports published in high-impact peer-reviewed journals (n=18), the professional press (n=21), four study reports and a doctoral thesis. The papers referred to research undertaken primarily in the USA (n=25), followed by the UK (n=12), Australia (n=5), Canada (n=1) and Iran (n=1). The papers were published between 2006 and 2017, with a peak in publication in 2012. The two earliest published papers (2006 and 2007) were authored by Meade, who was directly connected to the Studer Group, and these papers were heavily cited by authors publishing at later dates. The 44 papers were written by a total of 168 authors, with only 3 authors (Meade,^7 24 25^ Braide^6 26^ and Neville^27 28^) authoring or coauthoring more than one paper. This suggested there had not been a major programme of research or ongoing interest by one group of researchers in IR.

The programme theories for each of the eight mechanisms are presented below and then the CMOs summarised in table 4. It must be noted that these programme theories were not mutually exclusive, with one context and/or mechanism feeding into another or becoming an outcome of a third; however, they have been separated here for clarity. We will explore enabling contexts, which trigger or change the behaviour of these mechanisms, those contexts that act as barriers and how these CMO configurations may be linked, within the subsequent phase of this realist evaluation study.^15^

**Table 4:** Summary of eight context-mechanism-outcome (CMO) configurations

Mechanism title	Context	Mechanism (resources)	Mechanism (reasoning/responses)	Outcomes (from literature)
CMO 1: Consistency and comprehensiveness	Staged or simultaneous implementation approach Degree of fidelity to intervention Degree of adaptation and enhancement Staff education, training and understanding of intentional rounding Staff characteristics Risk/type of patient Workload issues/lack of time Presence of other organisational changes/competing initiatives	Intentional rounding helps keep patient care consistent through the use of a structured, systematic approach, ensuring all patient needs are met and potentially less obvious aspects of care are considered and managed at every round. Intentional rounding also helps ensure that family members are provided with consistent care and information in line with their needs (eg, the need for information, to be respected and to be comforted). It can also prompt agency staff to deliver care to a required standard.	This provides reassurance and confidence in the quality of care to patients, their family members and staff. This helps staff increase their vigilance and awareness of physical and psychological safety risks.	…and leads to higher patient and carer satisfaction with care and lower patient complaints. …and reduced patient uncertainty/anxiety. …and reduces call bell use. …and improves pain management. …and leads to a reduction in falls, pressure ulcers, bed-wetting and dehydration.
CMO 2: Allocated time	Embedding into existing daily routines Successful teamworking	Intentional rounding gives nurses allocated ‘time to care’ (ie, time to check that patients are comfortable and their needs are being met, thereby treating patients with dignity and replaces ‘presumed care’).	This helps nurses to organise their work and to feel able to prioritise this aspect of nursing care.	…and leads to nurses becoming empowered. …and to increased staff, patient and carer satisfaction with care and lower patient complaints.
CMO 3: Accountability	Design and suitability of intentional rounding documentation Leadership and management support Staff engagement and motivation Reason for implementation Who conducts the rounds?	Staff are required to complete and sign the intentional rounding record to say they have carried out hourly checks.	This makes staff feel personally accountable for the standard of care. This enables ward managers to monitor and audit the standard of care provided by nursing staff.	…and this leads to higher standards of care. …and provides a protection for both patients and staff as staff can evidence what they have done.
CMO 4: Nurse–patient relationships and communication	Ward setting/layout Staff characteristics Workload issues/lack of time	Intentional rounding provides increased and improved communication between staff, patients and family members, and ensures that patients’ perceived basic fundamental needs are met. It also provides more opportunities for positive nurse–patient relationships to develop based on trust, respect and caring.	This enables staff to get to know patients better and become more aware of their needs, and through this knowledge nurses can gather a keen sense of unusual behaviours and appearances and detect subtle/significant changes that can impact on comfort and safety.	…and this leads to a reduction in pressure ulcers, falls, bed-wetting and dehydration. …and improves pain management.
CMO 5: Visibility	Ward setting/layout	Intentional rounding increases the visibility/presence of nurses within a unit by increasing the time that nurses spend in the direct vicinity of their patients (ie, it gets nurses to the patient’s bedside).	This relieves the uncertainty and anxiety often associated with vulnerable patients’ hospital experience (ie, the inability to predict when care will be delivered and when someone will be available to assist them with care). This is comforting to family members because it denotes frequent and continuous assessment of the patient and their needs.	…and leads to improved patient and carer satisfaction with care and lower patient complaints. …and increases staff walking distances.
CMO 6: Anticipation	Type of patient Ward setting/layout	Intentional rounding enables nurses to anticipate/pre-empt and proactively address patient needs instead of being reactive and waiting for patient call bells and alarms.	This ensures that all patients receive regular care instead of unequally distributed care among patients, focused towards those who have frequent call bell use. Taking proactive and pre-emptive action reduces the likelihood of patients getting out of bed unattended.	…and this leads to a reduction in patient uncertainty/anxiety. …and increases patient confidence in staff. …and increases pain management. …and reduces call bell use, falls and pressure ulcers. …and contributes to a calmer ward environment with fewer interruptions to staff.
CMO 7: Staff teamwork and communication	Strong staff relationships Staff education, training and understanding of intentional rounding	Intentional rounding provides healthcare professionals with documented evidence.	This can be used to prioritise care in future rounds.	…and this leads to improved staff communication and teamwork.
CMO 8: Patient empowerment	Patient and carer education and understanding of intentional rounding Good staff–patient communication	Intentional rounding provides an opportunity for nursing staff, patients and family members to get to know each other better.	This empowers patients to ask for what they need in order to maintain their comfort and well-being.	…and this leads to higher patient and carer satisfaction with care.

### CMO 1: Intentional rounding ensures that consistent and comprehensive care is delivered to all patients by all nurses (n=21)

Twenty-one papers addressed this mechanism,^6 8 28–46^ highlighting the link between IR and consistent and comprehensive care. The structured, systematic approach to IR prompted and guided the delivery of care, helping staff remember to conduct all aspects of care on every round^38 41^ and identify tasks that might otherwise be missed.^36^ The format ensured continuity of care across staff members, helping guide junior/unqualified staff and those less familiar with patients.^38^ It also enabled persistent problems to be more readily identified, reducing the risk of repeating interventions that were not working.^6^ However, in most studies there was recognition that a dependence on routinisation and standardisation did not always ensure successful IR and that a flexible approach to process and design may be more appropriate.^28 31 33 34 43 46^ This involved nurses using their clinical judgement to modify the rounding process, assessing patients on an individual basis and making informed choices about how frequently to conduct rounds and what questions to ask.^6 28 30 33 34 38 42 44 45^ For example, there was evidence that nurses ‘rounded’ high-risk/high-need patients more and low-risk/low-need patients less regularly than rounding protocols stated.^28 33 34 37 42 44^ Setting of care was an important context for this mechanism, with reports that hourly rounds could be disproportionate for patients in mental health wards who had been assessed as low risk^38^ or intrusive for those experiencing psychotic symptoms.^42^ Other contexts that made IR more difficult included time limitations, low staffing levels and conflicting priorities.^28 39 40 44^

### CMO 2: Intentional rounding gives nurses allocated ‘time to care’ (n=19)

Nineteen papers addressed this mechanism^6 7 24 25 28 31 32 34 36 38–41 44 46–50^ and the original definition was only partially supported. There was no indication that nurses were given specifically allocated time in which to conduct IR. That is, there was no discussion of where nursing workload had been reduced or extra resources provided in order to assist with IR. However, there was some evidence that IR enabled nurses to manage their time more effectively, which could, in turn, free up more ‘time to care’.^36 38 40^ A reduction in call bell use following the introduction of IR was also reported to result in nurses having more time for patient care.^7^ No other descriptions of the mechanism in action were highlighted, and reported outcomes of the mechanism were limited, although it was proposed that the additional time generated by IR improved staff satisfaction,^7 24^ aided reflective practice^38^ and was satisfying to patients.^40^

There was more empirical evidence regarding the absence of the mechanism, with some staff reporting IR was ‘nothing new’, was what they were already doing or made little/no difference to their practice.^25 31 34 38 39 50^ Others believed IR resulted in *less* ‘time to care’ and increased their workload.^6 31 34 46 50^ Completing IR documentation was felt to take nurses away from patient care,^31^ with some having difficulty fitting IR around the rest of their workloads and ensuring it occurred at the correct time.^32 44 46^ In these situations, higher priority duties would take precedence over routine rounding^49^ and more complex patients prioritised over those deemed to be ‘well’.^6 28 44 48^ Few contexts associated with the mechanism were identified, although the need for cultural change to embed IR within existing routine practices within an organisation was perceived important to its success,^6 31 38 47 48^ as was successful teamworking.^28^

### CMO 3: Intentional rounding increases nurses’ accountability for the standard of care provided (n=19)

Nineteen papers addressed this mechanism^6 28 33–36 38–47 49–51^ and accountability (both personal and organisational) was perceived by some to underpin IR.^36 38 44 46 47 49^ However, the personal accountability of staff generally only related to responsibility for ensuring they completed IR documentation, rather than personal responsibility for carrying out high-quality rounds. Similarly, there were concerns that organisational audits of IR only provided information about staff compliance with documentation procedures rather than providing any record of rounding quality or confirmation that action(s) required had taken place.^6 38 39 41^ There were concerns that such audits provided incentives for staff to simply ‘tick boxes’ on the documentation rather than completing the tasks in full.^35^ For example, incidents were reported of nurses completing all documentation at the beginning/end of their shift^33 34 38 46 47^ or falsifying information on IR documentation when they had forgotten to complete it.^46^ The suitability of IR documentation was an important context for this mechanism, with evidence that, where documentation was not ‘fit for purpose’ or where rounding required nurses to complete additional paperwork, non-compliance with IR protocols was more likely to occur.^28 38–40 45^ Leadership and management support was also an influencing factor.^34 36 38 43–45^ Few studies discussed the outcomes of the mechanism, but some did report that nurses felt patronised, insulted or untrusted,^39 44 46 50^ while others felt IR devalued nursing work.^46^

### CMO 4: Intentional rounding enhances nurse–patient communication and/or relationships (n=17)

Seventeen papers addressed this mechanism^6 24 25 28 31 34 35 37 38 40 41 43 44 46 52–54^ and the original definition was partially supported. With regard to nurse–patient communication, it was widely reported that IR increased the amount of time nurses spent in direct contact with patients/family members and thereby increased the *frequency* of their communications.^25 31 38 40 41 43 52^ Staff believed that more frequent communications were welcomed by patients and family members,^6 25 31 37^ making them feel more involved in their care,^31^ empowered,^38 40^ more likely to voice concerns^25^ and less likely to feel ignored/neglected.^40^ However, there was less evidence that IR improved the *quality* of communications between nurses and patients/family members. Some staff felt using predetermined IR scripts stripped nurse–patient communications of authenticity and routinised patient contact.^46^ Some patients also highlighted the importance of quality interactions with staff, noting the importance of meaningful contacts and feeling connected.^35 40 53^ The second aspect of this mechanism related to nurse–patient relationships, and there was some evidence that these could be improved through IR. Some staff reported IR helped them get to know patients better,^40^ making them more aware of their needs,^25 31 34^ which could potentially impact on patient outcomes.^31^ It was felt this improved knowledge could also lead to better teamworking, enabling staff to hand over more detailed information about patients to the next shift.^40^

### CMO 5: Intentional rounding increases nurse visibility (n=11)

Eleven papers addressed this mechanism^6 25 26 31 34 36 40 41 43 44 48^ and some staff did believe IR increased the visibility of nurses on a unit.^25 34^ Increased visibility was generally viewed positively by nurses, with perceived benefits such as enhanced nurse–patient communication,^31^ helping patients feel well cared for and increased staff satisfaction.^43^ However, some negative outcomes were also reported, including an increase in non-urgent requests from patients.^48^ Some nurses questioned any association between IR and increased visibility, believing they were visible to patients even when they were not undertaking rounds. They could not, however, confirm whether this was also the patients’ perception.^40^ Of the studies that did explore patient perceptions, it was generally agreed that increased visibility of nursing staff was valued.^6 26 36^

### CMO 6: Intentional rounding enhances nurses’ ability to anticipate and proactively address patient needs (n=11)

Eleven papers addressed this mechanism^27 29 30 36–38 40 41 44 46 47^ and there was some empirical evidence to support the original definition. A number of staff stated IR enabled them to be proactive in anticipating patient needs, as opposed to being reactive to patient call bells/requests for help.^30 36 37 44 46 47^ This was associated with increased patient satisfaction^30^ and reassurance,^37 38^ decreased patient anxiety,^37^ a reduction in call bell usage,^38 40^ resulting in an overall sense of calm on the ward,^38^ and decreased staff workload.^46^ IR was also reported to enable nurses to intervene earlier when a patient’s medical condition was deteriorating, potentially preventing the need for higher levels of medical intervention^37^ and could prevent quieter patients being overlooked.^38 40 46^ Fewer studies reported patient experiences of the mechanism, although patient questionnaires demonstrated improvement in patient satisfaction^27^ and perceptions of nursing proactivity following the implementation of IR.^40^ One context associated with the mechanism was the type of patient and their particular needs. For example, changing position and getting in and out of bed were identified as activities that could be anticipated and addressed by IR but not pain management or toileting needs.^29^ The layout of the ward was also noted as an influencing context, with IR helping ensure patients in side rooms were not forgotten.^41^

### CMO 7: Intentional rounding enhances staff communication and/or teamworking (n=9)

Nine papers addressed this mechanism,^25 28 30 32 36 40 42–44^ with a number discussing the interconnecting relationship between staff communication and IR. That is, strong staff communication was perceived to be crucial for effective rounding, and rounding was perceived to improve staff communication.^32 36 40 42 43^ A similar relationship was also identified between IR and staff teamworking.^25 28 36 44^ When staff communication and teamworking were deemed to be ineffective, this caused frustration and concern among staff and reduced the effectiveness of IR.^28 44^ Contexts influencing the mechanism were staff involvement and ownership of practice^32 43^ and the busyness of the ward.^30^

### CMO 8: Intentional rounding fosters patient empowerment (n=4)

Four papers addressed this mechanism,^27 31 38 40^ although evidence was primarily drawn from one UK study.^38^ This reported patients becoming more ‘forthcoming’ when they knew staff were coming to see them regularly, empowering them to ask for what they needed. As in the original definition, this study also identified patient empowerment to be closely entwined with nurse–patient communication and relationships.^38^ Tentative evidence related to patient empowerment as an outcome of IR was identified by three studies.^27 31 40^

### Influencing contexts and outcomes of IR

While the aim of realist synthesis is to better understand the interplay of how a particular context has an impact on a specific mechanism to produce outcomes,^18^ the review highlighted that such detailed theoretical explanations are rarely provided in the IR literature. We elicited a list of potential ‘backdrops’ believed by authors to influence IR and a list of potential outcomes reported to arise from it. These are highlighted in tables 2 and 3, respectively. The findings of this synthesis echo those of a systematic review of the barriers to effective implementation and sustainment of IR on medical and surgical wards,^55^ as well as identifying additional ones. In table 4, we make explicit the theories by which IR may work by summarising the CMO configurations to be tested and refined in the subsequent realist evaluation phase of the study.^15^

## Discussion

In line with authoritative views on improvement science,^12 13^ our realist synthesis aimed to develop programme theories of IR to explain how, why, in what circumstances and for whom IR may or may not work. We interrogated the evidence in order to generate hypotheses, test and refine in the subsequent phases of our realist evaluation study. In a review of health improvement research in the UK, Dixon-Woods *et al* 56 argued that extensive development periods are needed to specify programme theory and select appropriate frameworks for analysis. It was notable that many studies included in the synthesis illustrate the absence of theoretical development as they only reported outcomes of implementing IR without providing any explanation of *how* or *why* they occurred. Similarly, many discussed the contexts that influenced IR but failed to explain how these conditions interacted with mechanisms to produce specific outcomes. This highlights the minimal theoretical explanations of IR that have been identified to date and the ambiguities surrounding its purpose. This poor understanding of how IR works poses a major challenge to learning, replication and sustainability of the intervention, and supports the work of others in this field.^11 56^

The synthesis identified a number of discrepancies between how IR is purported to work and how it operates in practice, as well as international differences in how the intervention has been implemented. For example, guidance from the USA states that the structured approach is a fundamental facet of IR and that the intervention should be used in a standardised manner so that all patients receive the same input.^2^ Yet other countries, including the UK, appear to have adopted a more flexible approach, based on nurses’ clinical judgement of patient need and preference. This is arguably because of differences in the drivers of implementation of IR in both countries. In the USA, IR is a marketed product,^2^ implemented in response to patient satisfaction surveys determining hospital financial reimbursement.^57^ However, in the UK, the Francis Inquiry was the major impetus for the widespread adoption of IR, with the aim of providing reassurance to the public at a time of intense media pressure. The UK government has been criticised for promoting IR and urging its introduction within NHS hospitals without being clear on what they were promoting and with no directives on how it should be undertaken or recorded.^3 9^ The intervention has therefore not been consistently implemented across settings but refined and adapted to suit local circumstances. It is not yet clear whether this flexible approach to IR has had a positive or negative impact on outcomes in the UK. What is clear, however, is that, both nationally and internationally, the intervention that one organisation refers to as IR is not the same as another, nor the same intervention that other empirical studies have evaluated. This leads to an important question of how flexible the approach to the delivery of IR can be before it can no longer be considered IR.

Another distinction between theory and practice of IR was highlighted in the ‘allocated time’ mechanism. While it has been claimed by some that IR would give nurses assigned time to care for patients’ basic needs, in the empirical literature, there was no evidence that nursing workload had been reduced or extra resources provided in order to assist nursing staff in carrying out IR. This is likely because, in practice, IR is not seen as a ‘time-requiring intervention’ but as fundamental care which should already be being done. It is little surprise, therefore, that many nurses felt IR was ‘nothing new’ or that it replicated what nursing staff had always done, but with added documentation. The ‘nurse-patient communication and relationships’ mechanism emphasised additional discrepancies by highlighting that IR may increase the *frequency* of nurse–patient communication, but not necessarily improve its *quality*. It is conceivable that increasing the frequency of nurse–patient communication alone could safeguard patients in settings where care delivery is poor. For example, reported instances of neglectful care, such as patients not being offered food, drink or pain relief for ‘many hours’ (point 7.314, p662),^58^ should not occur with an intervention that requires nurses to check these needs at hourly intervals. It is less apparent, however, whether simply increasing the frequency of nurse–patient communication would offer any further benefit to highly performing units or whether this might indeed reduce standards of care by taking nurses away from other key tasks and duties. This synthesis has established that, for many patients, increasing the frequency of communication alone is not enough and that it is the quality of their interactions with nurses that matters. This demonstrates the tensions around standardising nursing input within IR instead of accounting for the different needs of patients in order to provide interactions that are meaningful to them.

Managing risk has also been acknowledged as an important driver for the introduction of IR, and assumptions have been made that IR will increase the personal accountability of nurses and raise the overall standard of nursing care. However, this synthesis has identified that this is not necessarily the case. IR may assist organisations to monitor and audit the care provided by their nursing staff, but evidence suggests that these audits focus on compliance with documentation procedures rather than on the quality of rounds. Such focus on compliance with documentation is not necessarily a bad thing depending on whether or not the information gathered can be used by nurses to improve the care they deliver. However, there was evidence these audits provided incentives for staff to simply ‘tick boxes’ on the documentation, which could result in inaccurate records offering little protection to patients. This illustrates another ambiguity in the purpose of IR—is it to support nurses to improve the care they deliver, provide nursing managers with detailed evidence of nursing activity (eg, to support complaint handling), or is it an assurance tool for nurse directors seeking to report the quality of care to their boards and the public? This and other questions raised by this synthesis will be explored in subsequent phases of the upcoming realist evaluation in order to further refine and develop theories of IR.^15^

The weak evidence also did not give sufficient justification for the implementation of IR in the UK. Nevertheless, IR has been widely implemented and therefore the subsequent realist evaluation will focus on testing how contexts, mechanisms and outcomes of IR are configured.

### Strengths and limitations

This review has shown how IR may work by identifying the programme resources that the intervention offers and how these may be interpreted and acted upon by patients, carers and staff. It has specified how each of these mechanisms may or may not be triggered by different contextual factors and lead to a range of desired outcomes. The clinical outcomes included in this synthesis were specified in the protocol, for instance, call bell use, falls and pressure sores, but this review has also identified experiential outcomes, for example, empowerment, patient and carer satisfaction, and patient awareness of IR. Few studies included patient perspectives of IR.

The main limitation of this realist synthesis study was lack of robust evidence relating to IR and specifically the few insights about how IR was expected to work. The 35 of the 44 included studies which attempted this tended to be small, locally based evaluations, many of which relied on staff self-report or routinely collected data. IR outcomes included self-reported patient and staff outcomes, clinical outcomes, health economic and hospital-reported patient outcomes, but the predicted direction of change was not always clear-cut. Of the available evidence, only call bell use; patient, carer and staff satisfaction; pressure ulcers; patient falls; and patient complaint outcomes had a consistent pattern in the direction of change predicted by the majority of included studies, but these had serious methodological and/or reporting weaknesses, so should be treated with extreme caution.

## Conclusion

Despite the widespread use of IR, this paper highlights the paradox that there is ambiguity surrounding its purpose and limited evidence of how it works in practice. Our conclusions generate, first, understanding of how IR works in relation to context and outcomes; second, clarification of international differences in the drivers behind its implementation; and finally, learning for replication and sustainability of IR. Differences in the implementation of IR identified in the evidence demonstrate the importance of care delivery context and potentially that IR has been adapted in different contexts and over time.^16 59^ IR has been described as a ‘search for simple solutions to complex problems’,^60^ and it has been acknowledged that IR should not be perceived as a panacea to all problems.^61^ The findings of this synthesis agree with both points. It would appear, for example, that IR may well be an appropriate intervention to achieve particular goals (eg, increasing the visibility of nursing staff and frequency of nurse–patient communication) but less appropriate for others (eg, empowering patients or improving the meaningfulness of nurse–patient interactions). Further research is needed to test the eight a priori propositions about how IR may work, for whom, in what circumstances and why, and whether the investment of resources to support IR offers good value for money.
